# Pre-Exposure to Ionizing Radiation Stimulates DNA Double Strand Break End Resection, Promoting the Use of Homologous Recombination Repair

**DOI:** 10.1371/journal.pone.0122582

**Published:** 2015-03-31

**Authors:** Nakako Izumi Nakajima, Yoshihiko Hagiwara, Takahiro Oike, Ryuichi Okayasu, Takeshi Murakami, Takashi Nakano, Atsushi Shibata

**Affiliations:** 1 Advanced Scientific Research Leaders Development Unit, Gunma University, Maebashi, Gunma, Japan; 2 Research Center for Charged Particle Therapy and International Open Laboratory, National Institute of Radiological Sciences, Chiba, Japan; 3 Department of Radiation Oncology, Gunma University, Maebashi, Gunma, Japan; University Medical Center Hamburg-Eppendorf, GERMANY

## Abstract

The choice of DNA double strand break (DSB) repair pathway is determined at the stage of DSB end resection. Resection was proposed to control the balance between the two major DSB repair pathways, homologous recombination (HR) and non-homologous end joining (NHEJ). Here, we examined the regulation of DSB repair pathway choice at two-ended DSBs following ionizing radiation (IR) in G2 phase of the cell cycle. We found that cells pre-exposed to low-dose IR preferred to undergo HR following challenge IR in G2, whereas NHEJ repair kinetics in G1 were not affected by pre-IR treatment. Consistent with the increase in HR usage, the challenge IR induced Replication protein A (RPA) foci formation and RPA phosphorylation, a marker of resection, were enhanced by pre-IR. However, neither major DNA damage signals nor the status of core NHEJ proteins, which influence the choice of repair pathway, was significantly altered in pre-IR treated cells. Moreover, the increase in usage of HR due to pre-IR exposure was prevented by treatment with ATM inhibitor during the incubation period between pre-IR and challenge IR. Taken together, the results of our study suggest that the ATM-dependent damage response after pre-IR changes the cellular environment, possibly by regulating gene expression or post-transcriptional modifications in a manner that promotes resection.

## Introduction

Ionizing irradiation (IR) is widely utilized in cancer treatment, and the next generation of radiotherapy, including particle therapies, is promising [1]. However, exposure to IR can also increase the risk of cancer, although the risk associated with low-dose IR is still under debate [2,3]. Radiation-associated cancer risk is an important concern in environments such as outer space, where biological tissue can be exposed to relatively high doses of cosmic radiation.

DNA double strand breaks (DSBs) are potentially lethal to the cell if unrepaired, and their misrepair can give rise to genome instability [4]. DSBs can be repaired by either homologous recombination (HR) or non-homologous end joining (NHEJ). HR is a highly accurate system, whereas NHEJ frequently generates errors at repair junctions. Consistent with this notion, S/G2 cells are more radioresistant than G1 cells [5], and defects in HR exacerbate the incidence of cancer in breast and ovarian cancers [6,7]. The choice of DSB repair pathway is controlled by cell-cycle phase [8,9]. In G1, most IR-induced DSBs are repaired by NHEJ [10,11]. By contrast, HR becomes active in S/G2, although NHEJ is the predominant pathway throughout the cell cycle [12,13]. We previously showed that NHEJ initially attempts to repair DSBs in G2 (at two-ended DSBs, which are directly induced by IR); furthermore, if NHEJ does not ensue, the repair pathway switches from NHEJ to HR due to DNA damage or chromatin complexity [14]. HR is a multi-step reaction, of which DSB end resection is an early step [15]. Following the initiation of resection by MRE11 endonuclease activity, MRE11 3’-5’ exonuclease digests toward the DSB end, while exonuclease 1 (EXO1) generates ssDNA, moving 5’-3’ to create ssDNA coated with RPA [16,17]. Following resection by these exonucleases, BRCA2 replaces the RPA coat on ssDNA with RAD51. Next, RAD51-coated ssDNA invades the other DNA strand to generate a D-loop structure, which is required for DNA synthesis during HR. Subsequently, a crossover or non-crossover reaction completes the HR process.

Pre-exposure to low-dose IR (“pre-IR”) activates cellular defense systems in preparation for the next exposure to IR by accelerating DNA repair and antioxidant responses [18–20]. Numerous studies showed that pre-IR increases radioresistance and decreases chromosomal aberrations resulting from subsequent exposures to high-dose IR (“challenge IR”), a phenomenon referred to as the adaptive response. A recent report showed that neither induction of DSBs following challenge IR nor DSB repair kinetics in G1(G0) cells is affected by pre-IR exposure [21]. By contrast, in cycling cells, chronic pre-IR treatment promotes the usage of HR, as demonstrated by an HR reporter assay [22]. The observation of high rates of HR usage following pre-IR is consistent with the notion of increased radioresistance following pre-IR treatment. However, although it is evident that pre-IR promotes the usage of HR, it remains unclear which step in HR is upregulated by pre-IR.

In this study, we found that pre-IR treatment of G2 cells promoted the resection step of HR. Because the number of induced DSBs was unchanged, the percentage of DSBs subjected to end resection among all DSBs induced by challenge IR in G2 increased following pre-IR treatment, suggesting that the choice of DSB repair pathway was switched toward HR. Cells depleted for BRCA2, an essential HR protein, exhibited an additive DSB repair defect following pre-IR. This result supports the idea that the elevated fraction of DSBs undergoing resection as a result of pre-IR was repaired by BRCA2-dependent HR. Moreover, the elevated resection activity was diminished if cells were treated with a specific ATM inhibitor between pre-IR and challenge IR; this observation suggests that following pre-IR, ATM alters the cellular environment to bias the choice of repair pathway toward HR. This ATM-dependent modulation may be caused by changes in gene expression or post-transcriptional modification in response to IR. In summary, our study reveals that ATM-dependent upregulation of damage signaling by pre-IR stimulates DSB end-resection activity, promoting the usage of HR when cells are next exposed to challenge IR.

## Materials and Methods

### Cell culture and irradiation

A549 and H1299 lung carcinoma cells, U2OS human sarcoma cells, 1BR (wild-type) hTERT human fibroblasts, and 48BR (wild-type) primary human fibroblasts were cultured in Minimum Essential Medium (MEM) supplemented with 10% fetal calf serum (FCS) or Dulbecco's Modified Eagle's Medium (DMEM) supplemented with 15% FCS, 100 U/ml penicillin, and 100 μg/ml of streptomycin at 37°C in a humidified mixture of 95% air and 5% CO_2_. These cell lines were used in previous studies [17,23]. X-ray irradiation was performed at 200 kVp and 20 mA, using copper (0.5 mm)/aluminum (1.0 mm) filters, at a dose rate of 0.5 Gy/min. The ATM inhibitor KU55933 (Merck Chemicals) and the DNA-PKcs inhibitor NU7441 (TOCRIS) were added at a final concentration of 10 μM at the indicated time points; at this concentration, both drugs specifically inhibit their respective targets [14].

### siRNA knockdown

RNA interference was performed as previously described [14]. siControl and siBRCA2 were obtained from Dharmacon. siRNA was transfected into 1BR hTERT using HiPerFect (Qiagen).

### Homologous recombination and non-homologous end-joining assays

DSB repair assays were performed as previously described [17]. Direct repeat (DR)-GFP U2OS (1 × 10^5^ cells) for the detection of HR or H1299 dA3-1 (1.25 × 10^5^ cells) for the detection of NHEJ were plated in 6-well dishes 24 h before I-*Sce*I transfection. I-*Sce*I vector (p*Sce*I; 1.0 or 1.25 μg) was transfected using the GeneJuice or NanoJuice transfection kit (Novagen). After 48 h, cells were trypsinized, and GFP- or EGFP-positive cells were measured by FACS (FACSCanto; BD Biosciences) using the FACSDiva software.

### Immunofluorescence staining and foci scoring

Immunofluorescence (IF) staining was performed as described previously, and γH2AX and RPA foci were scored as previously described [14]. G2 cells were identified using the cell-cycle marker CENPF. To examine RPA foci in G2 phase in cycling cells, 4 μM aphidicolin (APH) was added immediately after the challenge IR. APH treatment blocks replicative polymerases and, hence, progression from S to G2 phase, and also induces a pan-nuclear γH2AX signal in S phase. γH2AX foci in G1 cells were examined by excluding cells with pan-nuclear γH2AX and CENPF signals. APH does not affect DSB repair (including RPA and γH2AX foci formation) in G2-phase cells [14,24]. For RPA, CENPF, and EdU triple staining, 10 μM of EdU was added 15 min prior to challenge IR. At the indicated time points, cells were pre-extracted and fixed with PFA. After fixation, cells were stained with EdU according to the protocol for Click-iT EdU Imaging Kits (Life Technologies). Following EdU staining, cells were stained with RPA and CENPF as described above. Microscopic images were taken by an Applied Precision DeltaVision OMX microscope using the settings for conventional image capture by a 100× objective. Approximately ten slices within 2–3 mm were taken as Z-series stacks. Images were stacked into a single-layer image without deconvolution.

### Antibodies for immunofluorescence and immunoblot studies

Antibodies against the following proteins were used for immunofluorescence (IF) and Western blotting (WB): γH2AX (05–636 JBW301; Upstate Biotechnology), CENPF (ab5; Abcam), RPA (NA-18, Ab-2; Calbiochem for IF, LS-C38952; LifeSpan BioSciences for WB), pRPA S4/8 (A300-245A; Bethyl Laboratories), pDNA-PKcs S2056 (ab18192), DNA-PKcs (12311; Cell Signaling), Ku80 (2180 (C48E7); Cell Signaling), pKAP-1 S824 (A300-767A; Bethyl Laboratories), KAP-1 (ab3831; Abcam), MRE11 (12D7, GTX70212; GeneTex), NBS1 (PC269, Ab-1; Oncogene), RAD50 (3427; Cell Signaling), pATM S1981 (2152–1; Epitomics), ATM (GTX70103 2C1; GeneTex), CtIP (A300-488A; Bethyl Laboratories), and β-tubulin (ab21058; Abcam). Ku70, XLF, XRCC4, and Ligase IV antibodies were kind gifts from Dr. Penny Jeggo. Immunoblotting was performed as described previously [17].

### Cell-cycle analysis by fluorescence-activated cell sorting (FACS)

Cells were fixed with 70% ethanol in phosphate-buffered saline (PBS) at the indicated time points. Fixed cells were washed with PBS and resuspended in PBS containing propidium iodide. Cell-cycle distribution was examined on a FACSCanto instrument using the FACSDiva software.

### Statistical analysis

Box plots were created from single experiments using SigmaPlot 12.0. Reproducible results were obtained from two or more independent experiments. Statistical significance was determined using Student's two-tailed *t*-test or the Mann–Whitney *U* test, also using SigmaPlot 12.0.

## Results

### Pre-IR treatment promotes the usage of HR, but not NHEJ, in a reporter system

**Fig 1:**
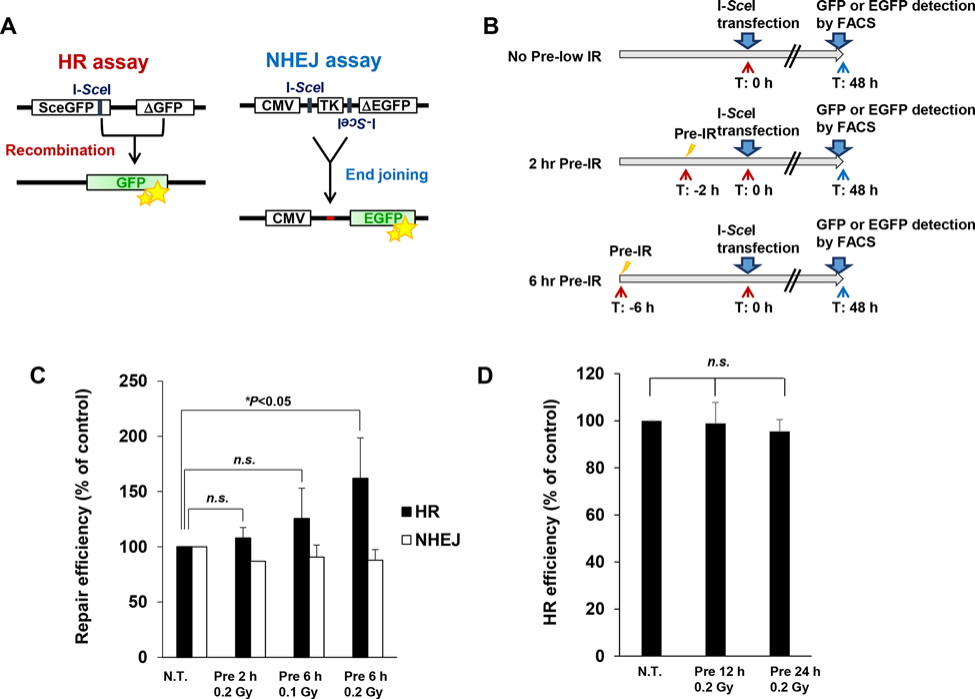
Pre-IR treatment promotes the usage of HR, but not NHEJ, in a reporter system. A) Summary of I-*Sce*I–based HR and NHEJ assays. HR and NHEJ assays were established as described previously. B) Schematic diagram of pre-IR treatment and I-*Sce*I induction in the repair assays. C) Pre-IR treatment increases the efficiency of HR. Cells were irradiated with 0.1 or 0.2 Gy X-rays 2 or 6 h prior to I-*Sce*I transfection, as shown in B. EGFP- or GFP-positive cells were quantitated by FACS 48 h after I-*Sce*I transfection. Error bars represent the SD of three independent experiments. D) Pre-IR treatment after 12–24 h does not affect HR efficiency. Cells were irradiated with 0.2 Gy X-rays 12 or 24 h prior to I-*Sce*I transfection. EGFP- or GFP-positive cells were quantitated by FACS 48 h after I-*Sce*I transfection. Error bars represent the SD of three independent experiments.

To investigate whether pre-IR treatment affects the balance of DSB repair-pathway choice between HR and NHEJ, we measured the efficiency of repair following pre-IR using chromosomal HR and NHEJ assays in the U2OS and H1299 cell lines, respectively (Fig 1A) [25,26]. To confirm that each assay monitored HR or NHEJ as predicted, we examined repair efficiencies in cells depleted for CtIP or BRCA2 using specific siRNAs or treated with a DNA-PKcs inhibitor. CtIP is essential for initiation of DSB end resection in HR [27], and BRCA2 plays an important role in HR by delivering and loading RAD51 onto single-stranded DNA. On the other hand, the kinase activity of DNA-PKcs is required for NHEJ. As expected, HR efficiency was significantly reduced in CtIP- or BRCA2-depleted cells, and NHEJ efficiency was decreased by the DNA-PKcs inhibitor, demonstrating that each assay is monitoring the desired pathway (HR or NHEJ), as expected (S1 Fig). To analyze the impact of pre-IR treatment on the choice of chromosomal DSB repair pathway, we treated cells with 0.1 or 0.2 Gy IR 2 or 6 h before I-*Sce*I induction (Fig 1B). Because irradiation doses greater than 0.5 Gy irradiation may lead to checkpoint arrest or cell death, in this study we treated cells with doses of 0.2 Gy or less (at these doses, no DSBs persist >6 h post-IR) [28]. Cells irradiated with 0.1 or 0.2 Gy IR 6 h prior to I-*Sce*I transfection exhibited elevated rates of HR, whereas pre-IR at 2 h or 12–24 h did not significantly affect HR (Fig 1C and 1D). Together, these results indicate that the rate of HR usage is elevated ~8 h after pre-IR treatment. The timing of pre-IR seemed to be important for this promotion of HR. On the other hand, the rate of NHEJ was slightly reduced by pre-IR, although this effect was not statistically significant (Fig 1C) (N.B.: because the efficiencies of HR and NHEJ were measured in separate assay systems, the rates of each type of repair are not linked).

### The additional amount of HR induced by pre-IR is BRCA2-dependent

**Fig 2:**
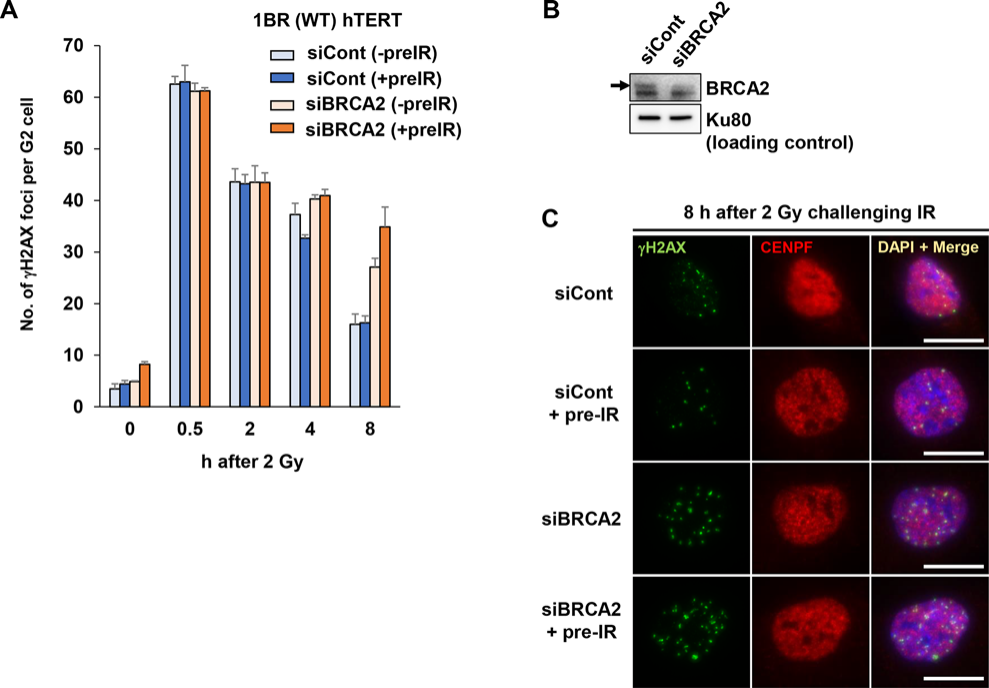
BRCA2-depleted cells exhibit an additive repair defect by pre-IR. A) Pre-IR treatment increases the fraction of BRCA2-knockdown cells with a DNA repair defect. 1BR hTERT cells treated with siControl or siBRCA2 were irradiated with 0.2 Gy X-rays 48 h after siRNA transfection. At 6 h after pre-IR, cells were irradiated with 2 Gy X-rays (challenge IR), and APH was added immediately after the challenge IR. γH2AX foci in G2 cells (CENPF+) were scored. Representative images of G2 cells are shown in S2A Fig. Error bars represent the SD of three independent experiments. B) Knockdown efficiency of BRCA2 is shown. Arrowhead indicates BRCA2. C) Representative images of the increase in the proportion of BRCA2-depleted cells with a DSB repair defect following pre-IR. Scale bar represent 10 μm (for all images).

To further validate the promotion of HR usage by pre-IR in cells exposed to challenge IR, we examined DSB repair in BRCA2 depleted cells, treated with or without pre-IR (Fig 2). Because BRCA2 is an essential HR protein that promotes RAD51 loading onto ssDNA following resection, loss of BRCA2 causes a defect in HR repair [29]. In addition, to investigate the regulation of DSB repair-pathway choice at a two-ended DSB, which is potentially repaired by HR or NHEJ, we examined DSB repair in challenge-irradiated G2 cells [30]. Consistent with our previous G2 data: without pre-IR, loss of BRCA2 resulted in a DSB repair defect relative to control cells at later time points (8 h) (Figs 2A–2C and S2) [13]. Importantly, BRCA2-depleted cells exhibited an additive DSB repair defect after 0.2 Gy pre-IR 6 h prior to the 2 Gy challenge IR, although the initial induction of DSBs was not affected at 30 min after the 2 Gy challenge IR (Fig 2A). These results suggest that pre-IR treatment increases the BRCA2-dependent HR fraction. Also, this result confirms our finding that the promotion of HR by pre-IR occurs not only in an endonuclease-dependent reporter assay, but also following challenge IR.

### DSB end resection after challenge IR is elevated in pre-IR–treated G2 cells

**Fig 3:**
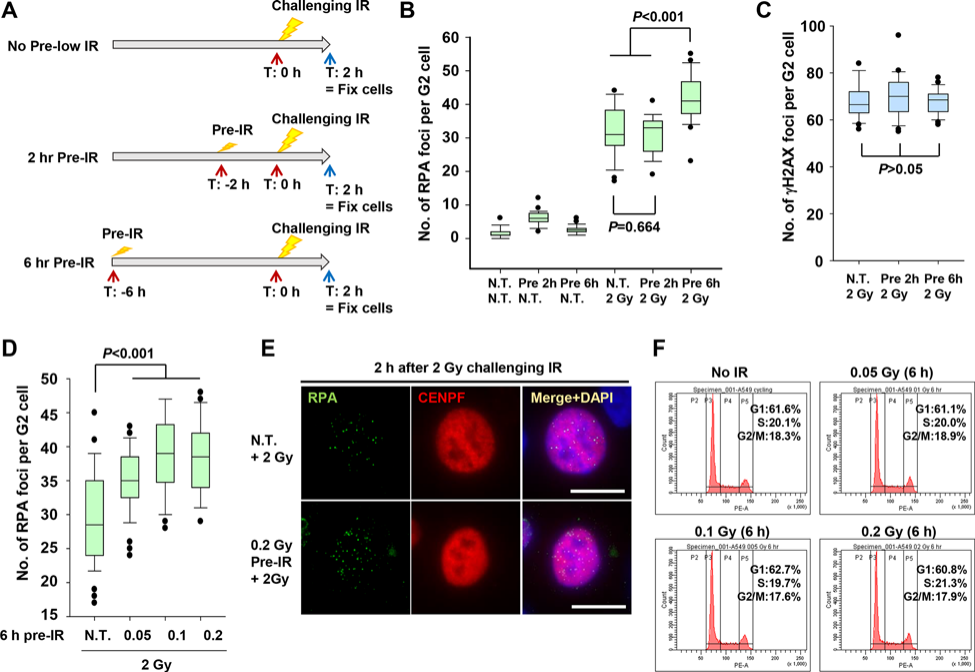
Pre-IR treatment promotes DSB end resection in G2. A) Schematic diagram of pre-IR treatment. Cells were irradiated with 0.2 Gy X-rays 2 or 6 h before a 2 Gy challenge IR. Cells were fixed and stained for RPA or γH2AX and CENPF 2 h after the challenge IR. APH was added immediately after the challenge IR. B) Pre-IR (0.2 Gy X-rays 6 h prior to challenge IR) promotes IR-induced RPA foci formation. RPA foci in A549 G2 cells (CENPF+) were examined 2 h after 2 Gy X-rays, with or without pre-IR (0.2 Gy X-rays) 2 or 6 h before the challenge IR. (N.B.: although there was a small increase in RPA foci number 2 h after pre-IR, there was no increase in RPA foci 6 h after pre-IR, i.e., the increase in RPA foci following 2 Gy preceded by pre-IR 6 h earlier is not due to damage persisting after the pre-IR treatment.) C) DSB induction is not affected by pre-IR. IR-induced DSB levels after a 2 Gy challenge IR in A549 G2 cells were examined by scoring γH2AX foci 30 min after IR. D) Dose of 0.05 Gy X-rays is sufficient to activate resection. A549 cells were irradiated with 0.05, 0.1, or 0.2 Gy X-rays 6 h prior to a 2 Gy challenge IR. RPA foci in A549 G2 cells were counted 2 h after 2 Gy challenge IR. E) Representative image of the increase in challenge IR-induced RPA foci following pre-IR. RPA foci in A549 G2 (CENPF+) cells are shown 2 h after the 2 Gy challenge IR; cells were exposed to 0.2 Gy pre-IR 6 h previously. Scale bar represent 10 μm (for all images). F) Dose of 0.05–0.2 Gy X-rays does not alter cell-cycle distribution. Cell-cycle distribution in A549 cells was examined by FACS 6 h after pre-IR.

To identify the specific step in HR repair that leads to elevated usage of HR, we investigated whether pre-IR treatment would increase DSB end resection in challenge-irradiated G2 cells. We focused on this step because resection is the most upstream event in HR, and it determines the choice of repair pathway [14,17]. First, to examine the timing of the effect by pre-IR, we irradiated A549 (p53+) cells 2 or 6 h (0.2 Gy) prior to a 2 Gy challenge IR (Fig 3A). To monitor the levels of DSB end resection, we counted RPA foci in A549 cells 2 h after the challenge IR (S2 Fig.). Because RPA binds to ssDNA following resection in irradiated G2 cells, IR-induced RPA foci represent resected DSBs undergoing HR. Importantly, RPA foci in irradiated G2 cells are CtIP/MRE11-dependent, suggesting that RPA foci formation is resection-dependent [13,14]. In these experiments, we used A549 cells, which form clearer RPA foci than primary or hTERT-expressing cells, but still exhibit normal repair responses [14]. To distinguish IR-induced two-ended DSB repair from replication-associated DSBs, which are predominantly repaired by HR in mid-S phase, and to avoid detection of resection-independent RPA foci in DNA replication, we focused on irradiated G2 cells by staining for CENPF, a G2 marker (S2 Fig.) [31–33]. As we previously reported, S phase cells exhibited pan-nuclear RPA (or γH2AX) signals that were enhanced by aphidicolin (APH) treatment (S2 Fig.).

Consistent with the elevated rate of HR observed in the experiments described above, the induction of RPA foci by challenge IR was higher in cells subjected to 0.2 Gy pre-IR (Fig 3B). This increase was not observed in cells pre-irradiated 2 h before the challenge IR, suggesting that the relevant cellular modulation requires >6 h. To confirm that pre-IR did not increase the total number of DSBs induced, we also counted γH2AX foci 30 min after the challenge IR in cells treated with or without pre-IR (Fig 3C). Importantly, induction of DSBs after the 2 Gy challenge IR was not altered by pre-IR, indicating that the elevation in the number of RPA foci following pre-IR treatment is caused by a repair-pathway switch in G2 cells. Next, to determine the lowest dose of pre-IR treatment that conferred these effects, we irradiated cells with three different doses. Pre-treatment with 0.1–0.2 Gy caused a significant increase in resection, whereas 0.05 Gy pre-IR mildly increased formation of RPA foci (Fig 3D; representative images are shown in Fig 3E). To further consolidate the increase in RPA foci in challenge-irradiated G2 cells, we stained cells with EdU/CENPF and RPA. Consistent with the results described above, 0.2 Gy pre-IR enhanced the number of RPA foci in CENPF^+^ EdU^-^ G2 cells after the challenge 2 Gy IR (S3 Fig.). Further, we confirmed that pre-IR treatment did not significantly change cell-cycle distribution (Fig 3F). In addition, we investigated RPA foci in normal human primary fibroblasts. A similar increase in resection was observed in response to 0.2 Gy pre-IR, although the magnitude of the increase was not as large as in cancer cells (S4 Fig.).

Taken together, these data show that pre-IR promotes DSB end resection in challenge-irradiated G2 cells without affecting the total number of DSBs induced, suggesting that the DSB repair pathway is switched from NHEJ to HR.

### Pre-IR promotes resection-dependent RPA phosphorylation at S4/8

**Fig 4:**
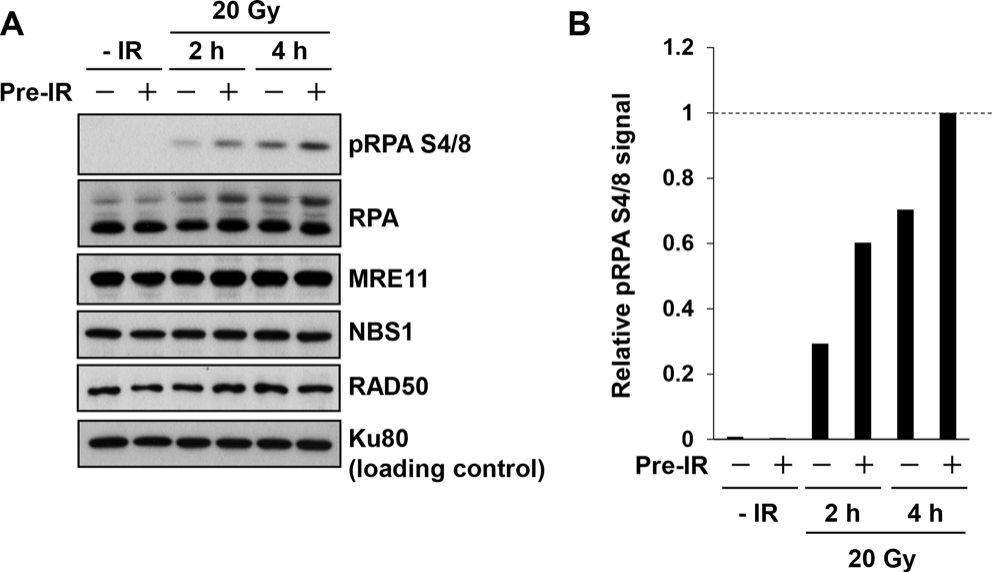
Pre-IR promotes resection-dependent RPA phosphorylation at S4/8. A) RPA Ser4/8 phosphorylation, which is correlated with levels of resection, is also promoted by pre-IR treatment. A549 cells were harvested 2 and 4 h after a challenge IR of 20 Gy; 6 h previously, cells were exposed to 0.2 Gy pre-IR (N.B.: due to limits on assay sensitivity, high-dose IR is required to detect RPA Ser4/8). The level of the MRE11–NBS1–RAD50 complex, which plays a central role in resection, was not altered by pre-IR treatment. Ku80, which is a NHEJ factor, was used as a loading control. B) pRPA levels were quantitated using ImageJ. The quantitation was performed on a single blot shown in panel A. Similar results were obtained in more than two independent experiments. Because the band representing total RPA is shifted due to phosphorylation, loading was normalized against the level of Ku80.

Next, we examined IR-induced RPA phosphorylation (pRPA) at serines 4 and 8 (S4/8), which are induced following resection [34]. At 2 and 4 h after the challenge IR, we observed an increase in RPA phosphorylation in cells treated with pre-IR (Fig 4A and 4B; N.B.: because of the limited sensitivity of the assay, >20 Gy IR is required to detect pRPA), confirming the elevation in the number of HR and RPA foci in response to pre-IR. Because the MRE11–NBS1–RAD50 (MRN) complex is a critical factor controlling resection, we also examined the expression levels of these proteins; however, we observed no significant changes (Fig 4A).

**Fig 5:**
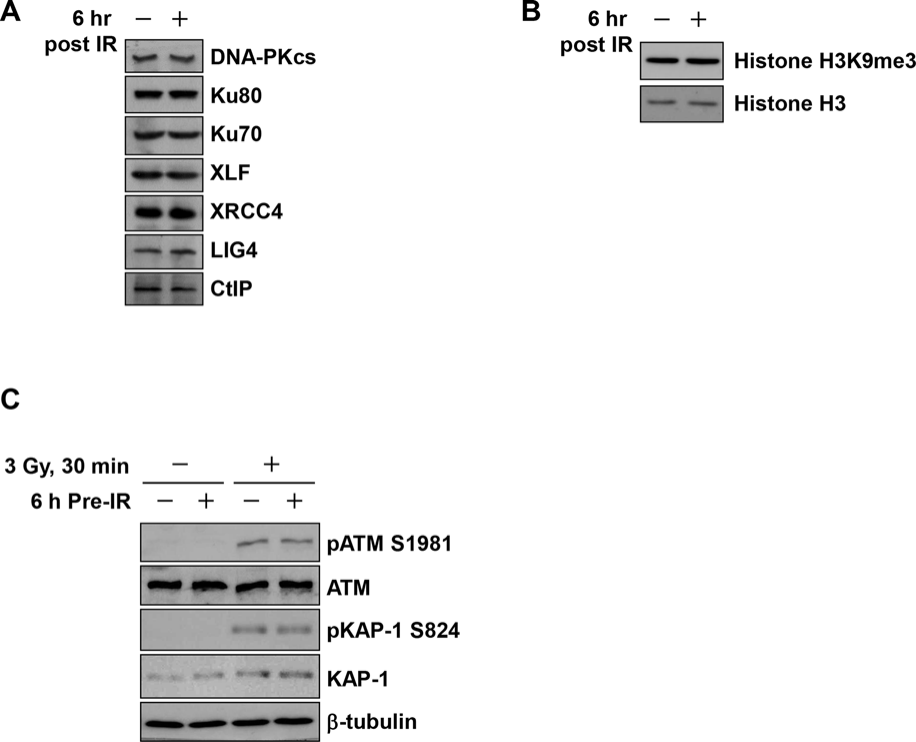
Analysis of IR-induced DNA damage signaling in cells subjected to pre-IR. A) The levels of NHEJ proteins did not change in response to pre-IR. A549 cells were harvested 6 h after 0.2 Gy pre-IR. CtIP, which is essential for resection, was also unaffected by pre-IR. B) Histone H3K9me3, which promotes the formation of heterochromatin, was examined in A549 cells 6 h after 0.2 Gy pre-IR. C) ATM signaling, ATM Ser1981 autophosphorylation, and downstream pKAP-1 Ser824 were examined 30 min after 3 Gy (challenge IR) in cells treated with or without 0.2 Gy pre-IR, followed by 6 h incubation.

Loss of NHEJ proteins promotes IR-induced DSB end resection [14]. Therefore, we examined the levels of NHEJ factors at 6 h following 0.2 Gy (the time point for the challenge IR); however, no significant alternations were detected by pre-IR (Fig 5A). The CtIP protein is required for initiation of HR [27]; however, its expression was not affected by 0.2 Gy pre-IR (Fig 5A). In addition, because heterochromatin influences the choice of DSB repair pathway, we also tested whether methylation status on histone H3 is altered by 0.2 Gy pre-IR [13,35]; however, we detected no change in global methylation levels (Fig 5B). To examine ATM activity and signaling, which also influence resection, we measured phosphorylation of ATM S1981 and KAP-1 S824 30 min after the challenge IR in cells treated with or without 0.2 Gy pre-IR [13,36]. However, ATM was activated normally irrespective of pre-IR treatment (Fig 5C).

### Enhanced resection by pre-IR treatment is ATM-dependent

ATM is the most important PI3 kinase required for activating the DNA damage response, which comprises DNA repair, signaling, checkpoint arrest, and apoptosis [37]. In response to DNA damage, ATM phosphorylates several hundred proteins [38]. Through multiple phosphorylation events, ATM-dependent signaling controls gene expression to regulate the DNA damage response following IR [39].

**Fig 6:**
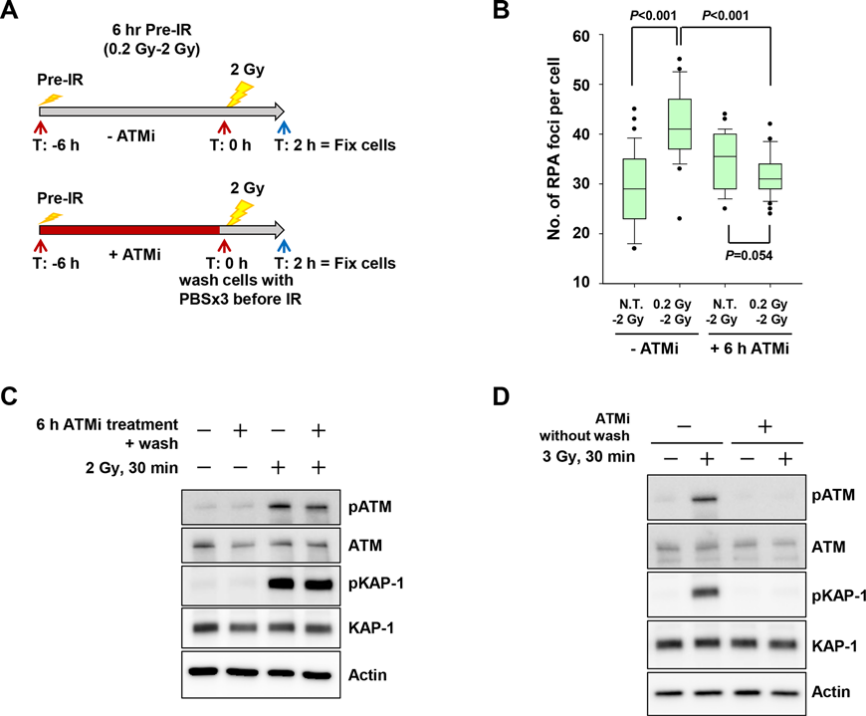
Promotion of resection by pre-IR treatment is ATM-dependent. A) Schematic diagram of pre-IR treatment with or without ATM inhibitor. A549 cells were irradiated with 0.2 Gy. 10 μM ATM inhibitor was immediately added after 0.2 Gy. After 6 h, fresh medium without ATM inhibitor was added following three washes with PBS. ATM activity recovered immediately following the removal of the inhibitor, as shown in panel C. B) Treatment with ATM inhibitor for 6 h attenuated the increase in RPA foci formation after pre-IR. RPA foci were counted in A549 G2 cells treated with or without 0.2 Gy pre-IR 6 h previously and with or without ATM inhibitor. C) ATM activity recovered following washing after 6 h treatment with ATM inhibitor. Phosphorylation of ATM S1981 and pKAP-1 S824 in A549 cells was examined 30 min after 2 Gy X-rays + 0.2 Gy pre-IR 6 h previously +/- ATM inhibitor treatment. D) Effect of ATM inhibitor was validated by detecting ATM autophosphorylation and KAP-1 phosphorylation. ATM inhibitor was added 15 min before 3 Gy challenge IR. Without removal of ATM inhibitor, A549 cells were harvested 30 min after the challenge IR.

We speculated that some cellular modulation, e.g., alterations in gene expression or post-transcriptional modifications of proteins associated with DSB repair, caused the increase in resection activity following pre-IR. To test this idea, we treated cells with a specific ATM inhibitor (ATMi) after pre-IR treatment. To suppress ATM-dependent cellular modulation by pre-IR, the inhibitor was added after pre-IR, but removed (by three rounds of washing) before the challenge IR. ATMi treatment attenuated the increase in resection following pre-IR (Fig 6A and 6B), suggesting that pre-IR may stimulate ATM-dependent changes in gene expression or post-transcriptional modifications that are necessary for promotion of DSB end resection. Recovery of ATM activity following removal of ATMi and the inhibitory effect of ATMi were confirmed by monitoring ATM autophosphorylation and KAP-1 phosphorylation (Fig 6C and 6D).

## Discussion

In this study, we demonstrated that pre-IR promotes DSB end resection, resulting in increased usage of HR repair. Previously, we demonstrated that NHEJ is the first choice in G2 cells following IR; however, if NHEJ does not rapidly progress due to DNA or chromatin complexity at DSB sites, the repair pathway switches from NHEJ to HR [14]. Conversely, if resection is not initiated, this switching does not occur, i.e., all DSBs in G2 are repaired by NHEJ without using the HR pathway at all. Thus, DSB end resection is a critical step that initiates and potentially commits the cell to repair by HR. Our observation that the number of DSBs undergoing resection is elevated following pre-IR is consistent with the notions we proposed in our previous study. We also examined factors involved in DSB repair, signaling, and chromatin, all of which may promote resection following pre-IR, but could not identify a specific factor responsible for this effect. A genome-wide gene expression analysis may help to identify such a factor; however, because gene expression is not always correlated with protein level or activity (due to post-transcriptional modifications), other approaches may be required to identify the relevant factor(s). This question will be addressed in future work. More importantly, we found that the increase in resection is ATM-dependent, suggesting that ATM-dependent damage signaling is required for activation of a factor that promotes resection following pre-IR. Expression levels of HDACs are elevated following IR [40]; because activated ATM changes chromatin structure by phosphorylation of KAP-1, which interacts with HDAC1/2, chromatin altered by ATM after pre-IR may generate a HR-prone environment.

γH2AX foci are well-defined DSB markers [30]. Foci analysis has several advantages, including high sensitivity and the ability to potentially detect all induced DSBs. However, because γH2AX foci represent all DSBs undergoing both NHEJ and HR, the rate of pathway usage cannot be estimated using this assay. Nevertheless, double staining of RPA (HR) and γH2AX (total DSB) is a useful tool for investigating the rate of HR usage after IR. Using this technique, we showed that pre-IR promoted resection/HR without affecting the overall induction of DSBs. To estimate the rate of NHEJ after IR, we subtracted the HR fraction from the total number of DSBs. DSB repair is conducted by not only NHEJ/HR, but also backup NHEJ pathway in human cells. However, we did not detect any obvious contribution from the backup NHEJ pathway in control G2 cells following IR (Shibata et al., in preparation), suggesting that the increase in HR was likely caused by a switch from NHEJ to HR. In this study, we did not see a significant reduction in NHEJ efficiency in the reporter assay (Fig 1). In irradiated G2 cells, the initiation of resection by MRE11 endonuclease activity directs the switch from NHEJ to HR [14]. Therefore, even a minor stimulation of resection may readily promote the switch from NHEJ to HR after IR. Conversely, because the NHEJ reporter assay picks up a specific event of NHEJ (rejoining two distant DSBs), a greater increase in resection activity may be required to promote the switch of repair pathway from NHEJ to HR.

Our observations are also supported by the concept of radioadaptation. Pre-IR prevents genomic instability, which leads to an elevated mutation frequency and tumorigenesis [41–43]. This effect could be explained by the promotion of HR, which is the most accurate repair pathway. However, although the elevated rate of HR usage could explain why cells treated with pre-IR become radioresistant, we hesitate to conclude that pre-IR increases overall genomic stability. Pre-IR shifts pathway choice from NHEJ to HR in G2; however, because normal cells must repair DSBs rapidly, NHEJ would still be preferable. HR is slow, and RAD51 recruitment can become saturated when large numbers of DSBs undergo HR [13,14]. In humans, the G2/M checkpoint is not as sensitive as the G1/S checkpoint [44]; therefore, when the redundant HR fraction forces cells to sustain G2, cells containing DSBs may progress into M due to leaks in the checkpoint, resulting in chromosome loss during mitosis. Thus, cell-cycle–dependence analysis will be necessary to assess the influence of pre-IR on genomic stability.

Similar to our observation, promotion of HR was also observed in low-dose–irradiated cells [22]. Yatagai et al. showed that low-dose IR prior to DSB induction led to an increase in HR, but not NHEJ by low-IR; however, note that this study used lower doses than those used in ours.

From a clinical standpoint, our results offer a novel rationale for X-ray radiotherapy. For instance, in some cancer cells under hypoxic conditions, HR is downregulated at the stage of RAD51 loading on chromatin, which is downstream of resection [45]. Because pre-IR promotes switching of the repair pathway toward HR, the switched fraction will not be repaired in HR-defective cells, i.e., pre-IR may increase hypoxia-induced tumor radioresistance. On the basis of our findings, we propose a novel radiotherapy protocol that would allow use of normal-dose radiotherapy against radioresistant tumors defective in HR.

## Conclusions

Our study revealed that the ATM-dependent damage response after pre-IR changes the cellular environment, leading to elevated usage of HR when cells are exposed to the next challenge IR. Future studies using microarrays or another type of screen are needed to elucidate the mechanism underlying the increase in resection by pre-IR.

## Supporting Information

S1 Fig Validation of the HR and NHEJ reporter assays. A) HR efficiency was measured following depletion of CtIP or BRCA2. siRNA knockdown was performed 48 h before I-*Sce*I transfection. GFP-positive cells were measured by FACS 48 h post I-*Sce*I transfection. CtIP is required for initiation of DSB end resection, BRCA2 is required for RAD51 loading onto ssDNA, and both of these steps are required for the completion of HR. Consistent with this, depletion of either CtIP or BRCA2 caused a significant reduction in HR efficiency. B, C) Knockdown efficiency of CtIP and BRCA2. Arrowhead in the right panel indicates BRCA2. D) Inhibition of DNA-PKcs by NU7441 reduces NHEJ efficiency. 10 μM NU7441 was added 8 h after I-*Sce*I transfection. After 48 h, EGFP-positive cells were measured by FACS.
(TIF)

S2 Fig Analysis of γH2AX and RPA foci in irradiated G2 cells. A) Representative image of γH2AX foci in 1BR hTERT cells 8 h after 2 Gy challenge IR. The strength of the nuclear CENPF signal increased from S to G2. CENPF-negative cells were in G1 phase. Mildly CENPF-positive cells with strong pan-nuclear γH2AX signals were in S phase. Pan-nuclear γH2AX signal in S phase was strengthened by treatment with APH. Strongly CENPF-positive cells with low γH2AX background signals were in G2 phase. B) Representative image of RPA foci in A549 cells 2 h after 2 Gy challenge IR. S-phase cells exhibited pan-nuclear RPA signals and moderate CENPF signals in a pattern similar to that of the γH2AX signal. By contrast, G2 cells exhibited clear RPA foci without a pan-nuclear RPA signal. RPA foci were not detected in G1 cells.
(TIF)

S3 Fig Increase in RPA foci following pre-IR in CENPF^+^/EdU^-^ G2 cells. To validate our finding that the number of RPA foci in G2 cells is elevated following pre-IR, we performed CENPF/EdU double staining. S-phase (CENPF^-^/EdU^+^) cells, which were not a focus of this study, contained pan-nuclear RPA signals. Conversely, A549 cells in G2 (CENPF^+^/EdU^-^) exhibited clear IR-induced RPA foci formation 2 h after 2 Gy challenge IR. Cells treated with 0.2 Gy pre-IR (6 h) contained more RPA foci than cells not exposed to pre-IR.
(TIF)

S4 Fig Pre-IR treatment promotes DSB end resection in primary human fibroblast cells. RPA foci in 48BR (WT) primary cells, treated with or without 0.2 Gy pre-IR (6 h), were examined 2 h after 2 Gy challenge.
(TIF)
