# Supplementary figures and images for: Pre-Exposure to Ionizing Radiation Stimulates DNA Double Strand Break End Resection, Promoting the Use of Homologous Recombination Repair
Source: PLoS One. 2015 Mar 31;10(3):e0122582. doi: 10.1371/journal.pone.0122582 (PMC4380452; doi:10.1371/journal.pone.0122582)

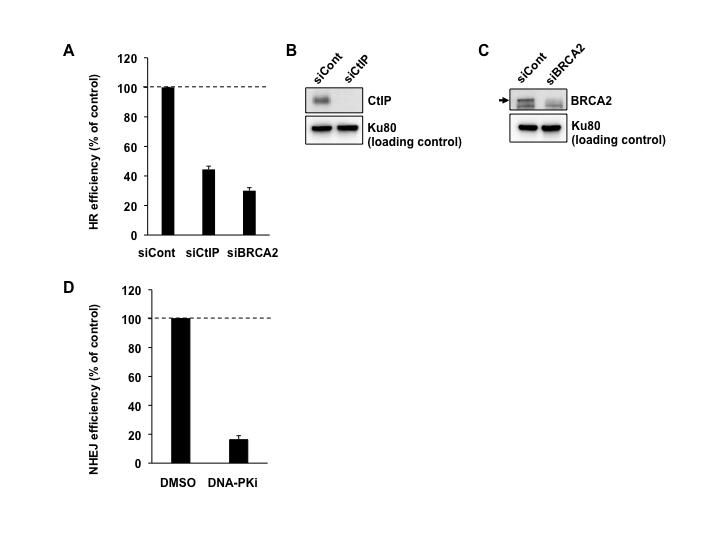

Supplement: S1 Fig — A) HR efficiency was measured following depletion of CtIP or BRCA2. siRNA knockdown was performed 48 h before I-SceI transfection. GFP-positive cells were measured by FACS 48 h post I-SceI transfection. CtIP is required for initiation of DSB end resection, BRCA2 is required for RAD51 loading onto ssDNA, and both of these steps are required for the completion of HR. Consistent with this, depletion of either CtIP or BRCA2 caused a significant reduction in HR efficiency. B, C) Knockdown efficiency of CtIP and BRCA2. Arrowhead in the right panel indicates BRCA2. D) Inhibition of DNA-PKcs by NU7441 reduces NHEJ efficiency. 10 μM NU7441 was added 8 h after I-SceI transfection. After 48 h, EGFP-positive cells were measured by FACS. (TIF) [file pone.0122582.s001.tif]

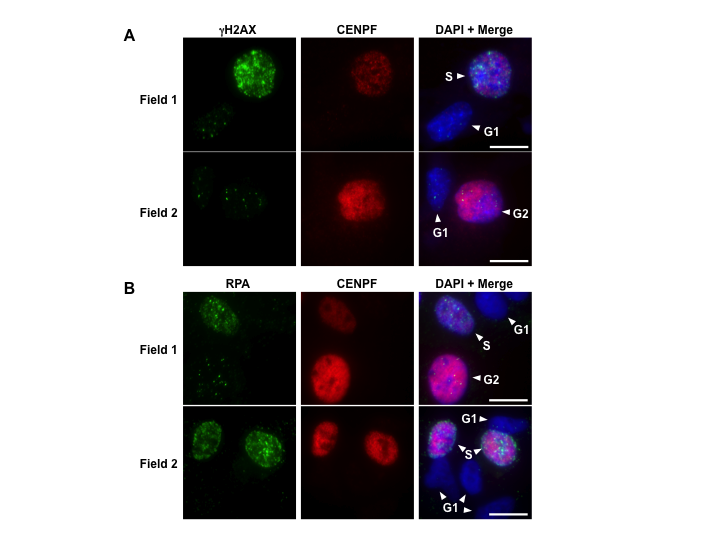

Supplement: S2 Fig — A) Representative image of γH2AX foci in 1BR hTERT cells 8 h after 2 Gy challenge IR. The strength of the nuclear CENPF signal increased from S to G2. CENPF-negative cells were in G1 phase. Mildly CENPF-positive cells with strong pan-nuclear γH2AX signals were in S phase. Pan-nuclear γH2AX signal in S phase was strengthened by treatment with APH. Strongly CENPF-positive cells with low γH2AX background signals were in G2 phase. B) Representative image of RPA foci in A549 cells 2 h after 2 Gy challenge IR. S-phase cells exhibited pan-nuclear RPA signals and moderate CENPF signals in a pattern similar to that of the γH2AX signal. By contrast, G2 cells exhibited clear RPA foci without a pan-nuclear RPA signal. RPA foci were not detected in G1 cells. (TIF) [file pone.0122582.s002.tif]

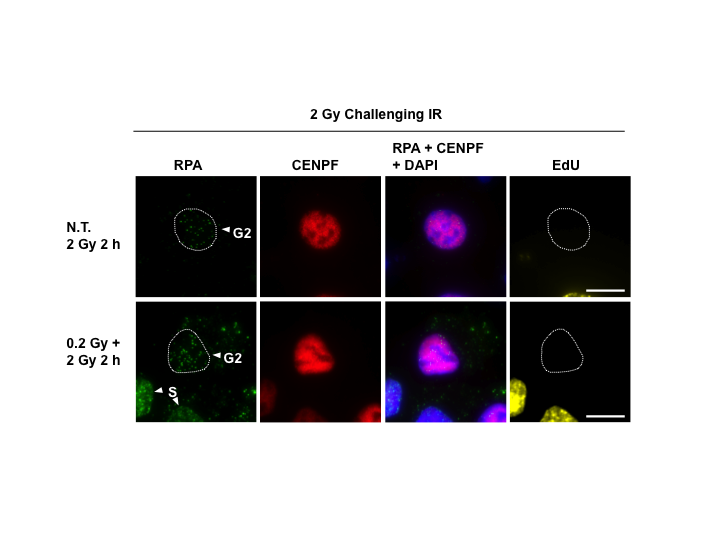

Supplement: S3 Fig — To validate our finding that the number of RPA foci in G2 cells is elevated following pre-IR, we performed CENPF/EdU double staining. S-phase (CENPF-/EdU+) cells, which were not a focus of this study, contained pan-nuclear RPA signals. Conversely, A549 cells in G2 (CENPF+/EdU-) exhibited clear IR-induced RPA foci formation 2 h after 2 Gy challenge IR. Cells treated with 0.2 Gy pre-IR (6 h) contained more RPA foci than cells not exposed to pre-IR. (TIF) [file pone.0122582.s003.tif]

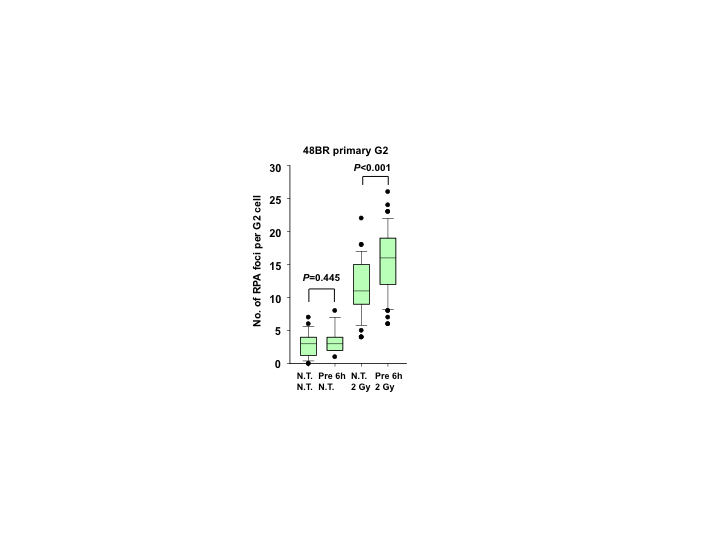

Supplement: S4 Fig — RPA foci in 48BR (WT) primary cells, treated with or without 0.2 Gy pre-IR (6 h), were examined 2 h after 2 Gy challenge. (TIF) [file pone.0122582.s004.tif]
